# The impact of stance during heel raises on the hybrid ultimate lifting kit (HULK) device: A future microgravity exercise machine

**DOI:** 10.3389/fphys.2022.943443

**Published:** 2022-08-23

**Authors:** Logan Kluis, Ravi Patel, William K. Thompson, Beth Lewandowski, Ana Diaz-Artiles

**Affiliations:** ^1^ Department of Aerospace Engineering, Texas A&M University, College Station, TX, United States; ^2^ Sibley School of Mechanical and Aerospace Engineering, Cornell University, Ithaca, NY, United States; ^3^ NASA Glenn, Cleveland, OH, United States; ^4^ Department of Health and Kinesiology, Texas A&M University, College Station, TX, United States

**Keywords:** biomechanics, opensim®, kinematics, dynamics, microgravity

## Abstract

Extended missions in microgravity, such as those on the International Space Station (ISS) or future missions to Mars, can result in the physiological deconditioning of astronauts. Current mitigation strategies include a regimented diet in addition to resistance training paired with aerobic exercise. With the increased effort toward long duration space missions, there is room to optimize the cost, required time of use, and mass of exercise equipment. This research effort focuses on understanding the biomechanics of Heel Raise (HR) exercises while using the Hybrid Ultimate Lifting Kit (HULK) device, an exercise device designed to optimize volume and functionality. Using the biomechanics tool OpenSim, the effect of HR foot stance (15° inward, 15° outward, and straight) was assessed by analyzing kinematic and kinetic data. In particular, we analyzed peak joint angles, range of motion, joint moments, and angular impulses of a single subject. Preliminary results indicated no significant differences in terms of ankle/metatarsophalangeal/subtalar joint angles, range of motion, joint moments, and angular impulses between foot stances. In addition, loaded HR exercises were compared to body weight HR exercises without the HULK device. Finally, recommendations are made towards an optimal HR routine for long-duration space missions. The impact to health and rehabilitation on Earth is also discussed.

## Introduction

Future spaceflight missions to the Moon and Mars will require extended travel times to and from the mission locations (Mars Architecture Steering Group, 2009). As a result, astronauts will be exposed to long periods of microgravity that can lead to physiological complications impacting the success of these missions. Specifically, the crew will experience the deconditioning of the neurovestibular, cardiovascular, and musculoskeletal systems. For the purpose of this research effort, a focus will be placed on mitigating the well-documented negative effect of prolonged microgravity on the musculoskeletal system (West, 2000; Trappe et al., 2009; Grimm et al., 2016). For example, microgravity has been known to decrease the volume of some muscles by up to 10.3% in as little as 8 days (LeBlanc et al., 1995), or up to 15.4% in 16 days (Akima et al., 2000). The muscles of the calf, (i.e., the gastrocnemius and soleus) have reduced in volume by an average of 17% after longer duration missions, and once back on Earth, these losses can take up to 60 days to recover (LeBlanc et al., 2000). In addition, bone loss can occur at a rate 10 times greater than that of post-menopausal women (Cavanagh, Licata and Rice, 2007), and it is estimated to be up to 1% per month in some cases (Nicogossian et al., 2016). These physiological decrements are magnified when considering that astronauts are expected to perform EVAs upon landing on Mars.

One of the main mitigation strategies to limit the loss of bone and muscle mass is the implementation of exercise protocols (Smith et al., 2012). Due to the weightless environment, creative methods to provide resistive exercise to the crewmembers are imperative. The Hybrid Ultimate Lifting Kit (HULK) device developed by ZIN Technologies is a spaceflight exercise device prototype that utilizes gas cylinders and electric motors to provide resistance to the user (Thompson et al., 2015). The ability to perform squats and deadlifts on the HULK device has previously been studied (Thompson et al., 2019), but the HULK’s impact on heel raises is yet unknown. Heel raises will be part of any spaceflight exercise routine and the ability for users to confidently perform them on the HULK device will be critical. Variations in foot stance during heel raise exercises results in changes in muscle activation of the muscles in the lower leg (Riemann et al., 2011; Cibulka et al., 2017), which could be integrated into a training program. In this research effort, we specifically focus on investigating lower body kinematics and dynamics as it relates to foot stance when performing heel raise exercises on the HULK device.

## Methods

### Testing setup and heel raise exercise configurations

The data for the study were collected from a 68 kg male subject at NASA Glenn Research Center. All testing was done with approval from the Institutional Review Board at NASA Glenn and informed consent from the subject. The subject performed heel raises with the HULK device in four configurations. The first three configurations consisted of heel raises in different foot stances with resistance provided by a t-bar that the subject held with his two hands in front of him. In all three configurations, the resistance transmitted by the t-bar was 75 kg (165lbs). The three different foot stances were as follows: 15° rotated outward, 15° rotated inward, and 0° (i.e., feet straight). In addition to these three configurations, an additional control configuration of heel raises was performed with just body weight as resistance (i.e., no t-bar), and with a straight stance (i.e., feet at 0°) which was designated straight stance without HULK (WH). In this condition, the subject balanced without touching the HULK device (i.e., without external support or load). Each configuration was performed for multiple repetitions, which were averaged for analysis. The number of good quality repetitions in each configuration varied between 3 and 5. Specifically, 5 repetitions were successfully captured during the 15-degree outward and 0-degree configurations, 4 repetitions were successfully captured during the 15-degree inward configuration, and 3 repetitions were successfully captured during the straight stance without the HULK device. A good quality repetition was determined based on the time to complete the repetition (which was approximately 1 s), full kinematic range of motion, and the completeness of the data from which the repetition was taken.

**FIGURE 1:**
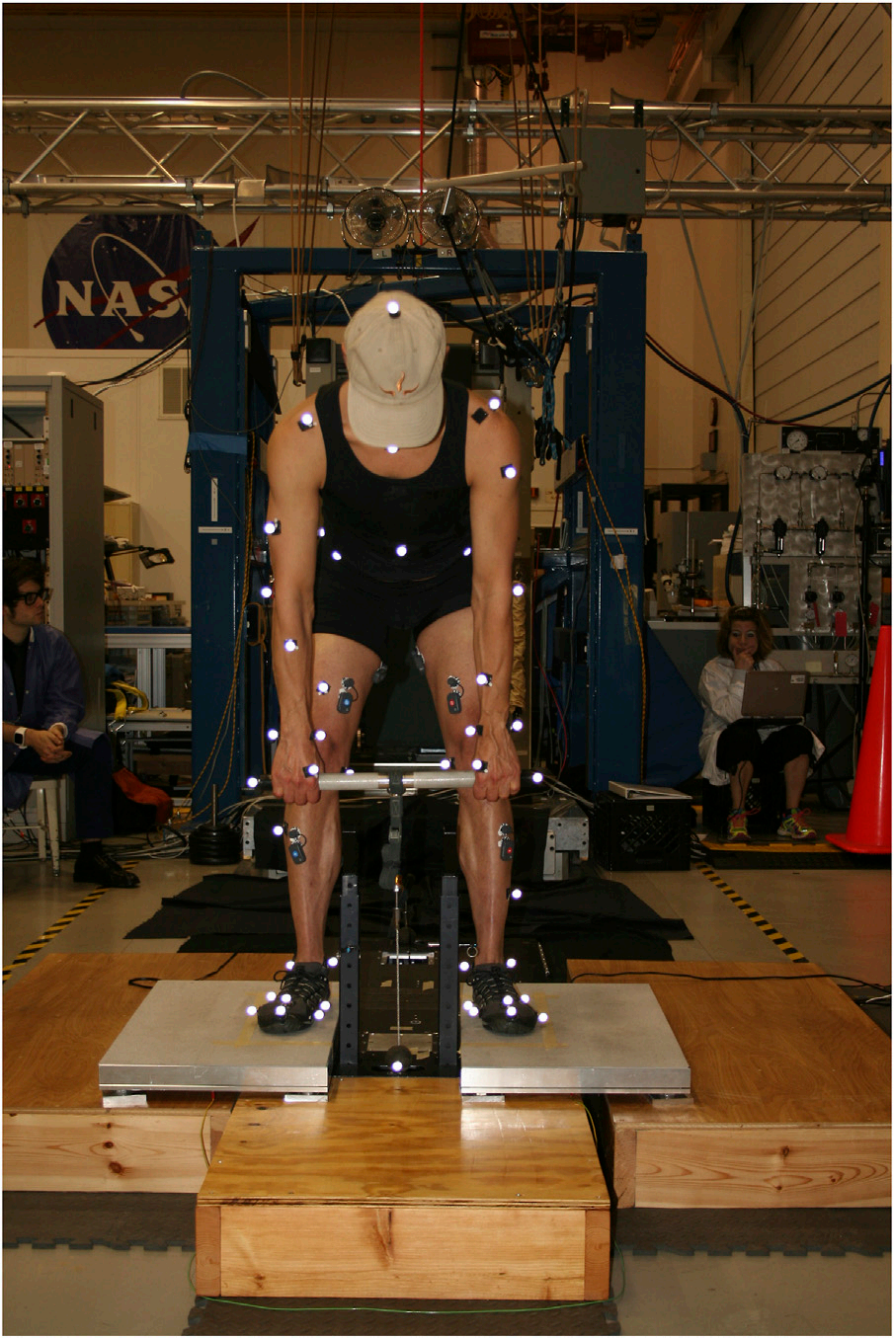
The HULK testing setup. The subject is holding the t-bar of the HULK device. In addition, the motion capture markers are seen covering the upper and lower body.

Motion data of the heel raises were recorded in all configurations using a Smart DX System (BTS Bioengineering, Quincy, MA) at 100 Hz. The motion capture system tracked the movement of 59 reflective markers placed at specific locations on the subject’s body and t-bar while performing the heel raises. In addition, force plate data were obtained to record ground reaction forces at 200 Hz which was synchronized with motion capture data. The HULK contained load cells in line with the t-bar cable for measuring the resistance during the exercises. The load cell measurements were also synced with the motion capture and force data. An image of a subject with motion capture markers using the HULK device can be seen in Figure 1.

**FIGURE 2:**
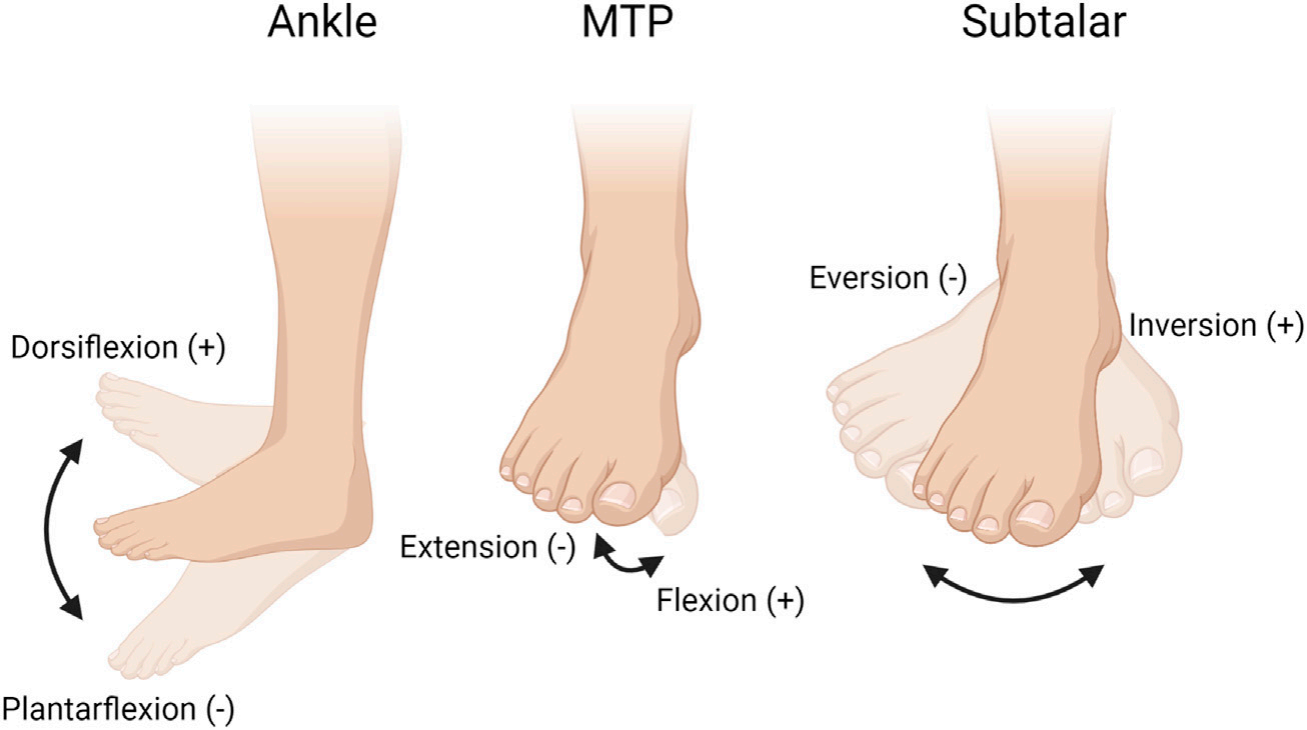
The three joints analyzed during the heel raises exercise: the ankle joint (dorsiflexion/plantarflexion), the metatarsophalangeal (MTP) joint (flexion/extension), and the subtalar joint (inversion/eversion). Figure created with BioRender.com.

For the present analysis, we focused on the three joints located within the foot: the ankle joint (dorsiflexion/plantarflexion), the metatarsophalangeal (MTP) joint (flexion/extension), and the subtalar joint (inversion/eversion). These movements are shown in Figure 2. Specific dependent variables include joint angles (including range of motion and peak angle), and joint moments (including angular impulse and peak moment) during each HR configuration. Only the right foot was used for the analysis of angles and moments. Finally, the center of pressure throughout the HR repetitions was also calculated.

### OpenSim simulations

To process the motion capture data, we used a biomechanics software called OpenSim. OpenSim has a large variety of capabilities such as inverse kinematics (IK) and inverse dynamics (ID). In addition, the software has proven capable of assessing a variety of motions such as walking (Anderson and Pandy, 2001), running (Hamner, Seth and Delp, 2010), jumping (Anderson and Pandy, 1999), and squatting. The software has also been used to model the effect of spacesuit joint torques (Gilkey, 2012; Diaz and Newman, 2014) and their impact to extravehicular activity (Kluis et al., 2021a; Kluis et al., 2021b). The human model used for our simulations was a full body model developed by Apoorva Rajagopal and modified by researchers at NASA Glenn Research Center (Hicks et al., 2015; Rajagopal et al., 2016). In addition to the human body, a geometry file was loaded into the model to represent the HULK t-bar, which is where the resulting force from the HULK device was applied.

The first step to the analysis consisted of scaling the OpenSim model to match the size of our subject. Using spatial marker data from the motion capture data, we adjusted the anthropometric measurements of our model to match those of the subject. In addition, the mass of the model was adjusted to 68 Kg to match the mass of our subject. With appropriate anthropometric measurements, additional steps were performed to ensure a proper connection of the HULK t-bar (which was also modeled in OpenSim) to the hands of the model, allowing the transfer of forces from the HULK resistive cable to the human model. To accomplish this, two additional markers were added to the t-bar, and these were leveraged to create a point and ball joint between the t-bar and the left and right hands of the scaled OpenSim model, respectively.

In the next step, we performed inverse kinematics to calculate joint angles based on the collected motion capture data. The noise associated with our experimental data acquisition was first reduced using a Butterworth filter (cutoff frequency 6 Hz). OpenSim calculates the inverse kinematics by minimizing the (weighted) squared errors between the experimental motion capture markers and a series of virtual markers previously placed on the model. Given the purpose of the study and our specific interests, the markers located on the lower body of the model were given a higher weighting scheme (×50). In addition, the markers located around the HULK TBAR were also weighted higher (×25) to ensure a proper connection between the TBAR and the hands of the model. In the last step, based on the previously calculated kinematics solutions and incorporating the foot and TBAR external forces, we performed inverse dynamics to calculate the net forces and moments at every joint in the model produced during the heel raise exercise.

### Data processing and statistical analysis

**FIGURE 3:**
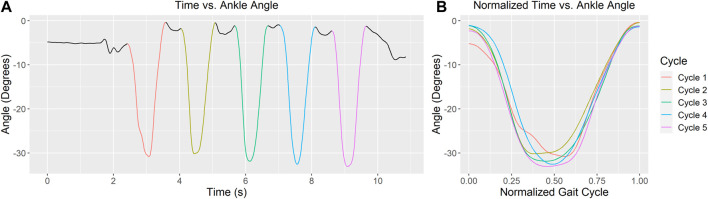
**(A)** Ankle angle during straight foot stance heel raises over time (5 repetitions). Each repetition is marked with a different color. **(B)** Normalized repetitions are superimposed for further analysis.

The joint angles and moments for the right ankle, MTP, and subtalar joints were visually inspected to ensure appropriate quality for further analysis. For each configuration, valid HR repetitions were normalized (to 101 samples, from 0 (the start of the repetition) to 1 (the end of the repetition), averaged, and used for analysis. This process is visually represented in Figure 3.

To compare the stance positions and effects of the HULK device to straight stance repetitions, peak joint angles, range of motion, peak moments, and angular impulses were assessed statistically. First, the data were tested for normality (Shapiro-Wilk test) and heteroscedasticity (Breusch-Pagan test). Some of the data did not comply with these assumptions, and therefore we implemented non-parametric techniques. In particular, a non-parametric Mann-Whitney *U* test was used to compare the inward, outward, and straight foot stances. Similarly, the Mann-Whitney *U* test was also used to compare the straight foot stance with and without the HULK device. A Mann-Whitney *U* test was chosen over a statistical test for dependent variables because we considered that the individual repetitions of a given stance (e.g., first repetition of the straight stance) are not necessarily dependent on the individual repetitions of any other stance (e.g., first repetition of the outward stance). As a result, and acknowledging the limitations related to the small sample size, we chose to use an independent variable statistical test. Significance was set to α = 0.05. When comparing the three stance conditions (with HULK), a Bonferroni correction for multiple comparisons was implemented and, in this case, α = 0.05/3.

In addition to the analysis of the biomechanics global metrics above, we also investigated differences in kinematics and dynamics during the time course of the HR repetition between the three different foot stances (inward, outward, straight). To compare stances, we performed pairwise comparisons at each normalized time point using a non-parametric Mann-Whitney *U* test. If two stances presented statistically significant differences of at least 10 consecutive time normalized points (*p* < 0.05), then that phase of the heel raise was considered to be significantly different between the foot stances. This method was used in place of an overly conservative Bonferroni adjustment because of the elevated number of pairwise comparisons conducted (101 pairwise comparison, which would yield an α = 0.05/101). In addition, this method of analysis was already used in previous HULK studies (Thompson et al., 2019).

Finally, we analyzed the center of pressure of the subject’s contact with the force plates. Center of pressure assessments are used to confirm correct placement of the feet through the entire exercise period and identify abnormalities in subject movements. The center of pressures for the left and right feet through the entire exercise period of a given condition were plotted and assessed qualitatively with visual inspection.

## Results

### Overall metrics: Peak angle, range of motion, peak moment, and angular impulse

**FIGURE 4:**
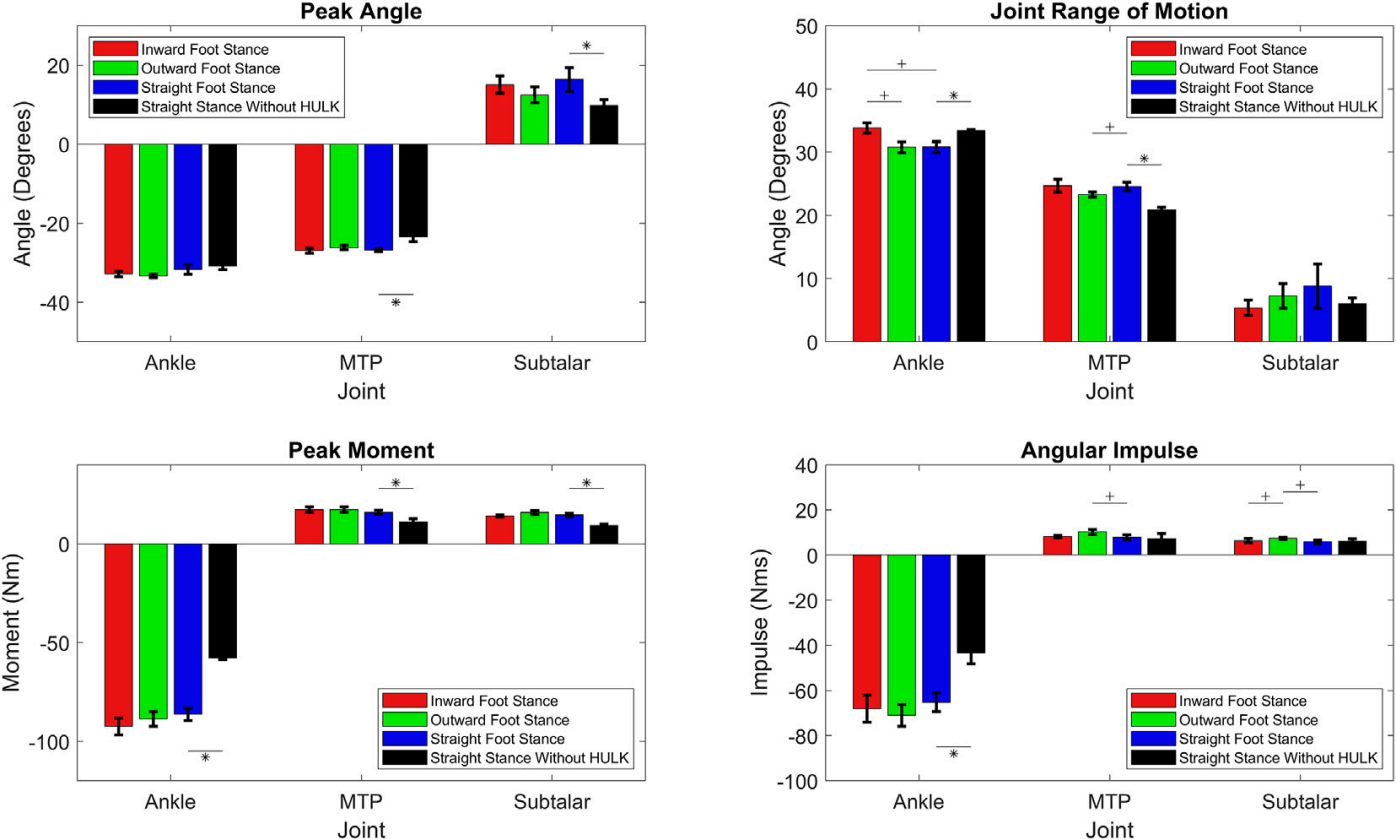
Peak angle, range of motion, angular impulse, and peak moment in each one of the three joints investigated (ankle, MTP, subtalar) for inward foot stance, outward foot stance, straight foot stance, and straight stance without HULK conditions. A “+” symbol indicates significant differences between an inward, outward, or straight foot stance (α = 0.05/3), and a “*” symbol indicates significant differences between straight foot stance and straight stance without HULK (α = 0.05). Data presented as average ±SD.

Figure 4 summarizes the peak angle, range of motion, peak moment, and angular impulse results for the three joints investigated (ankle, MTP, and subtalar) in each one of the 4 conditions (inward, outward, straight, and straight without HULK). Quantitative values are also summarized in Table A1. Overall, these metrics indicate only minimal changes to kinematics and dynamics because of foot stance with HULK loading. Only a small portion of the joints displayed significant statistical differences. Specifically, most of the statistical difference can be found in the range of motion and angular impulse. For example, there is a significant difference between the ankle range of motions of inward and outward foot stances (*p* = 0.0159) and inward and straight foot stances (*p* = 0.0159). The outward and straight foot stance’s range of motion are significantly different in the MTP joint (*p* = 0.0159). Similarly, the outward and straight foot stances (*p* = 0.0159) and the inward and outward foot stances (*p* = 0.0159) have significantly different angular impulses in the Subtalar joint. Finally, the angular impulse of the outward and straight stance in the MTP joint are significantly different (*p* = 0.0159).

Analysis of the straight foot stance with and without the HULK device resulted in numerous metrics that are significantly different. The ankle joint has significantly different range of motion (*p* = 0.036), peak moment (*p* = 0.036), and angular impulse (*p* = 0.036). The MTP joint has significantly different peak angle (*p* = 0.036), range of motion (*p* = 0.036), and peak moment (*p* = 0.036). Finally, the subtalar joint has significantly different peak angle (*p* = 0.036) and peak moment (*p* = 0.036).

### Biomechanical differences in normalized heel raise cycle between outward, inward, and straight foot stances using the HULK device

**FIGURE 5:**
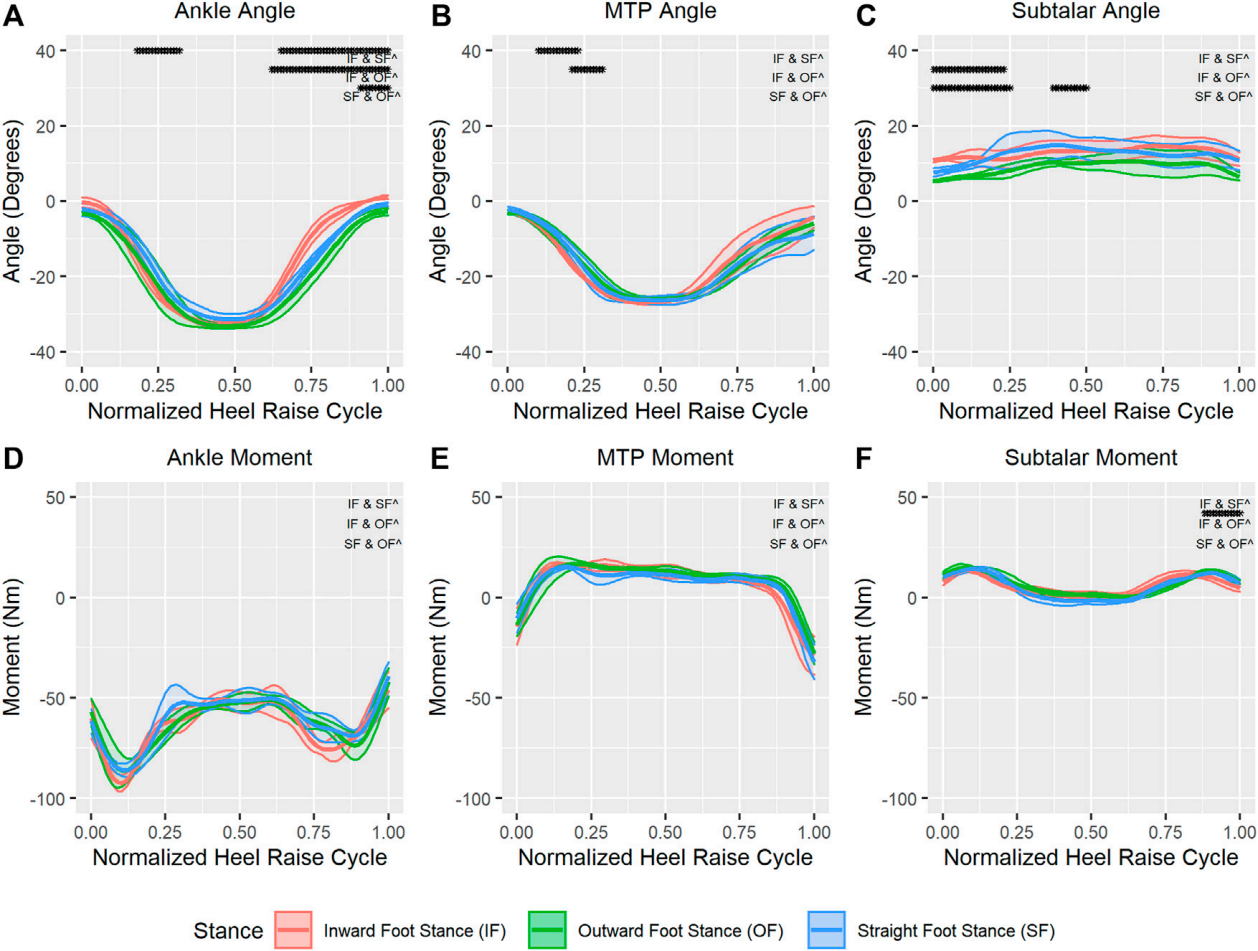
Average (±SD) ankle joint angle **(A)** and moment **(D)**, MTP joint angle **(B)** and moment **(E)**, and subtalar joint angle **(C)** and moment **(F)** for outward, inward, and straight foot stances through a normalized heel raise cycle. Solid center lines indicate means, and the surrounding shaded areas indicate ±1 standard deviation. Asterisks indicate statistical significance (α = 0.05) through a phase of a minimum of 10 consecutive statistically significant time normalized points.

The top of Figure 5 shows the average (±SD) joint angles for the ankle (dorsi/plantarflexion), MTP (flexion/extension), and subtalar (inversion/eversion) joints during HR exercises in the three stances considered (outward, inwards, and straight). For the ankle in Figure 5A, most of the statistically different phases are located at the end of the repetition for all three comparisons. In addition, there is a small region approximately at the 25% of the HR cycle where we found differences between the inward foot stance and straight foot stance. This region represents the ascending phase of the heel raise. Similarly, the MTP angle in Figure 5B shows significant differences between the inward stance and the outward and straight stances at approximately 25% of the HR cycle. Finally, the majority of the subtalar joint angle in Figure 5C shows differences between stances only in the first 25% of the HR cycle, specifically between the outward foot stance and the inward and straight foot stances. In addition, there is also a short phase close to the peak of the HR where we also found significant differences between the outward and inward foot stances.

The bottom of Figure 5 shows the average (±SD) moments for the ankle (Figure 5D), MTP (Figure 5E), and subtalar joints (Figure 5F) during HR exercises in the three foot stances considered (outward, inwards, and straight). In general, none of the joints showed any significant differences between foot stances in the required moments for the HR exercise. One exception is the subtalar moment, were we found differences between the outward and inward foot stance between the 80%–100% of the HR cycle.

### Biomechanical differences in normalized heel raise cycle with straight foot stance with and without the HULK device

**FIGURE 6:**
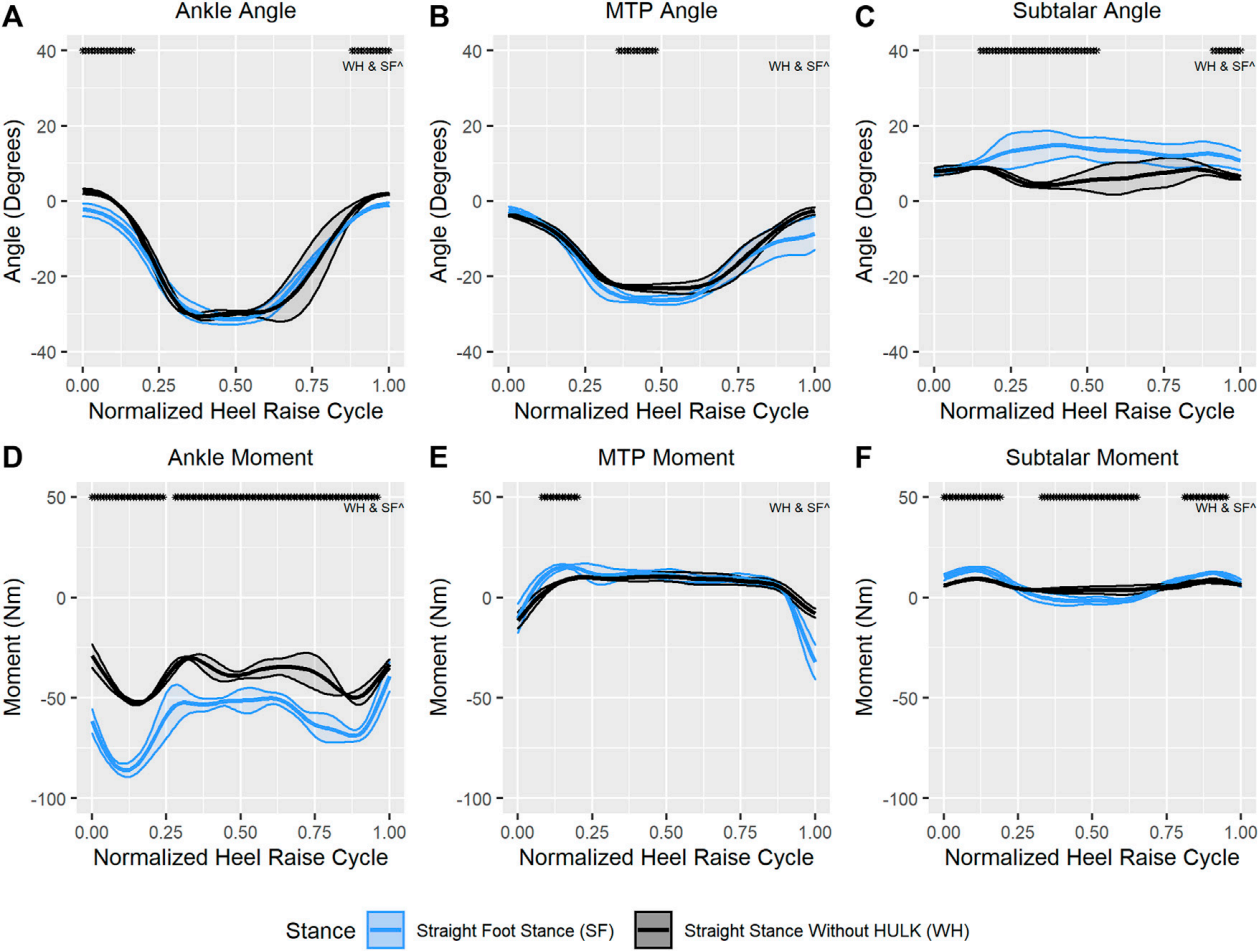
Average (±SD) ankle joint angle **(A)** and moment **(D)**, MTP joint angle **(B)** and moment **(E)**, and subtalar joint angle **(C)** and moment **(F)** for straight foot stance and straight stance without HULK conditions through a normalized heel raise cycle. Solid center lines indicate means, and the surrounding shaded areas indicate ±1 standard deviation. Asterisks indicate statistical significance (α = 0.05) through a phase of a minimum of 10 consecutive statistically significant time normalized points.

The top Figure 6 shows the average (±SD) ankle (Figure 6A), MTP (Figure 6B), and subtalar joint angles (Figure 6C) during the HR repetitions for the straight foot stance with the HULK device and for the straight stance without the HULK device. The beginning and end phases of the ankle joint during a HR cycle showed significant differences. In comparison, the MTP angle was only significantly different between the two configurations at approximately the 50% mark of the HR cycle. Finally, the subtalar joint angle showed significant differences between the straight foot stance with and without the HULK device through the raising phase and the end phase of the heel raise cycle.

The bottom of Figure 6 shows the average (±SD) moments for the ankle (Figure 6D), MTP (Figure 6E), and subtalar joints (Figure 6F) during the HR repetitions for the straight foot stance with and without the HULK device. As expected, given the difference in resistance, the majority of the ankle and subtalar moments were significantly different between the two conditions. Specifically, the ankle moment showed significant differences almost throughout the entire HR cycle, except for a small number of time-normalized points at the 25% mark and at the very end of the cycle. Similarly, the subtalar moment exhibited significant differences between both configurations throughout the entire heel raise cycle, except for a few points at the 25% and 75%, marks, as well as at the very end of the cycle. Finally, and in contrast to the other two joints, the MTP moment only presented significant differences between the two conditions during a short period of the HR cycle around the 13% mark.

### Center of pressure

**FIGURE 7:**
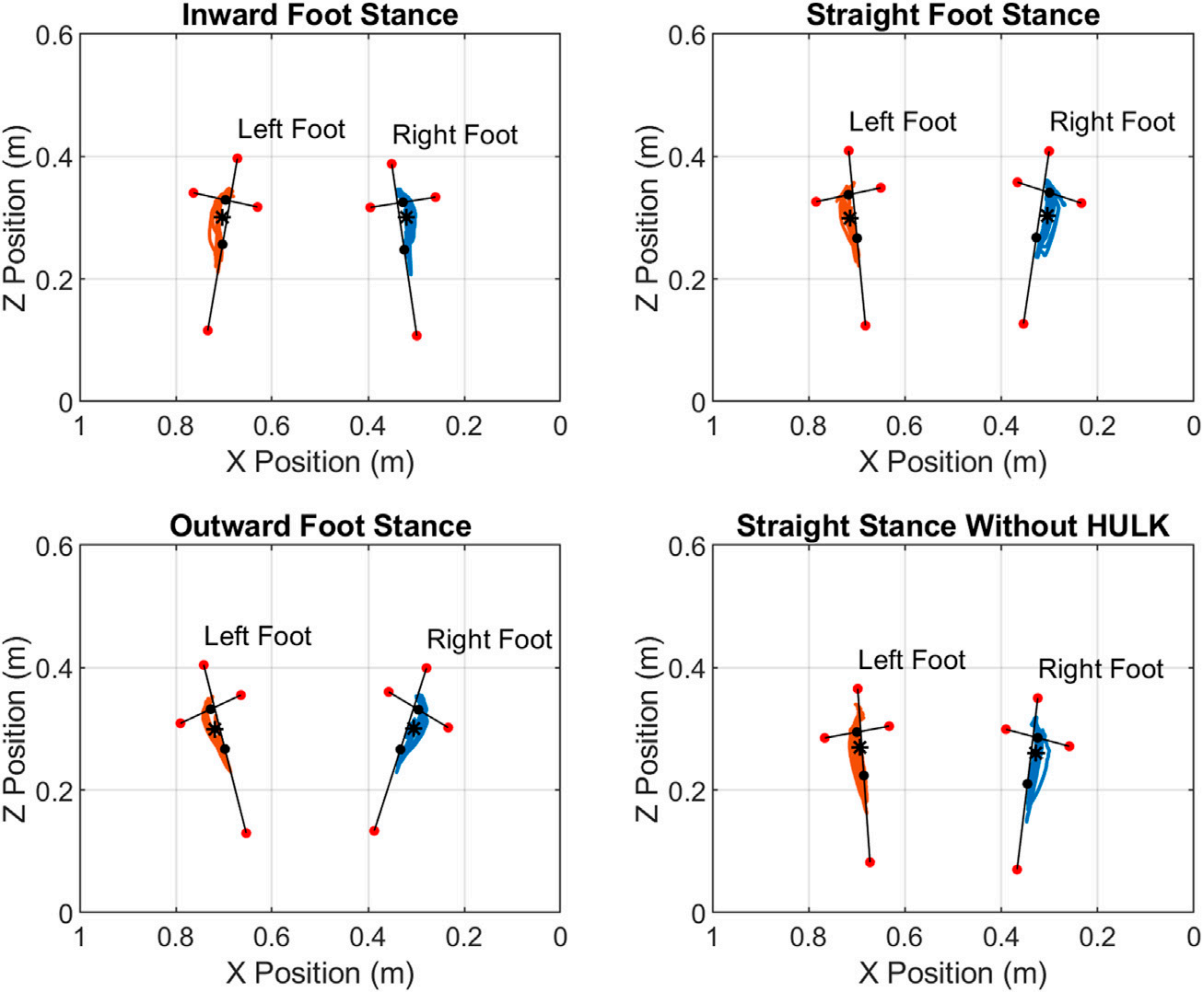
Center of Pressure during the heel raise repetitions for the four different configurations investigated: inward, straight, and outward foot stances with the HULK device, and straight stance without the HULK device. The blue line indicates the center of pressure of the right foot, and the orange line indicates the center of pressure of the left foot. Red dots indicate the location of motion capture markers on the toe, heel, outside, and inside of the left and right feet. Black dots indicate center points of the toe/heel line and the inside/outside line. The asterisks represent the centroid of the center of pressure through the entire set of HR repetitions. Note: the x axis has its origin on the right side of the figures and increases towards the left. This is due to the origin of the force plates being located in the bottom right corner.

Figure 7 shows the progression of the center of pressure through every heel raise cycle for the left and right foot in all three stances with the HULK device and straight stance without the HULK device. Four markers were located on each foot, and their location is also shown in the figures. Finally, center points for the inside/outside markers and toe/heel markers are shown next to the centroids of the center of pressure. In general, the center of pressure maps look as expected, with the centroid for each stance being located toward the “ball” of the foot. The figures are also useful for visually comparing the magnitude of difference in stance positions.

## Discussion

The overall metrics for the three stances with the HULK device have differences in only the range of motion and angular impulse. Specifically for the range of motion, the inward foot stance is significantly different than the outward and straight stances in the ankle joint, and the outward foot stance is significantly different than the straight stance in the MTP joint. For angular impulse, the outward foot stance is significantly different than the inward and straight stances in the MTP joint, and the outward stance is significantly different than the straight stance in the subtalar angle. The small number of significantly different metrics over all three stances with the HULK device are indicative of the minimal impact that stance has on the peak angle, range of motion, peak moment, and angular impulse of the joints in the lower leg during HR exercises. In comparison, the straight stance with and without the HULK device indicated a significant impact from the HULK device to the peak angle, range of motion, peak moment, and angular impulse metrics of the subject. This was an expected result as additional resistance to the exercise will add forces and moment throughout the lower body, and as a result, impact the overall kinematics of the exercise.

Generally, we found minor changes in kinematics and dynamics between stances. The majority of the statistically significant differences in the kinematic cycles can be identified at the beginning and end of the HR exercise cycle. This is likely due to the choice of repetition start and end time and not a result of the stance change. For example, lengthening the entire repetition time by choosing an earlier overall start time can result in an initial angle that affects the mean and standard deviation for the given time normalized point. This issue is unavoidable as any choice of start or end point will have impacts to the overall kinematic curve of the stance and joint combination. As previously specified, preference was given to matching peak kinematic angles as these were assumed to be more sensitive to the impact of stance change. While the joint kinematics have several areas of statistical difference, the joint moment had notably few significantly different phases. In particular, the inward and straight stances for the subtalar joint had different moments during the end phase of the heel raise cycle. Like the kinematic curves, this can be contributed to the choice of the end-of-cycle time point. Finally, change in stance had very little impact on the location of the center of pressure centroid relative to the stance of the feet. The three stances with the HULK and straight stance without HULK share visually similar hysteresis paths for their center of pressures.

As can be predicted, there were significant differences between the kinematics and dynamics of straight stance heel raises with and without the HULK device. The most prominent difference in the kinematics appears in subtalar joint at the peak of the heel raise. The straight stance without HULK repetitions displayed much less inversion than with the HULK. It is likely that the additional force the HULK device applies to the lower body requires greater recruitment from the muscles, which alters the kinematics of the exercise. This claim is supported by the large change in moment in the ankle and subtalar joint. The ankle, for example, had a larger resistive moment throughout the entire movement to maintain stability at higher loads, which will inevitably require larger muscle activations. Interestingly, the subtalar joint moment remains relatively constant throughout the heel raise cycle in the condition without the HULK, while the subtalar moment with the HULK generates a sinusoidal-like curve. This is certainly a consequence of the higher load and the change in kinematic angles that arise from this additional load.

Future spaceflight missions will inevitably include heel raises as an exercise in the astronaut’s routine. If equipment similar to the HULK device is available, our results indicate that the stance of the astronaut will most likely not have any significant impact on the kinematics or dynamics of their HR lift. This information could be useful if certain joint angles or moment limits are trying to be avoided to minimized injury risk. These results indicate that users should select a stance that is the most comfortable and/or targets a desired lower-body muscle group. For example, studies used EMGs to identify that the medial gastrocnemius activates more than the lateral gastrocnemius when the subject performs the exercise with an outward foot stance (Riemann et al., 2011; Cibulka et al., 2017). When an inward foot stance is used, the lateral gastrocnemius activates more than the medial gastrocnemius. Also, when the subject performs heel raises with increased flexion in the knee, there are increases in activation of the soleus muscle with respect to the medial and lateral gastrocnemius muscles (Signorile et al., 2002). With known activation of the soleus and medial and lateral gastrocnemius, a workout program can be designed to target the desired muscle or muscle groups that are most affected by microgravity by altering foot stance. A combination of targeted muscle activation and proper muscle loading will allow for improvements in muscle mass in the lower leg muscles. The choice of foot stance will be one part of a comprehensive workout program to mitigate the loss of gastrocnemius and soleus muscle mass. In summary, the change in foot position during heel raises on the HULK device does not affect the kinematics and dynamics but based on previous studies (Riemann et al., 2011; Cibulka et al., 2017), we hypothesize that it does affect the activation of the gastrocnemius and soleus. This can be coupled with proper loading and exercise programs to create an effective spaceflight-induced muscle loss countermeasure. Finally, this research is applicable and informative for Earth-based exercise and rehabilitation. Like astronaut exercise regimes, lower body rehabilitation plans frequently incorporate heel raise exercises to strengthen muscle groups in the lower legs. Depending on the injury, certain foot stances during the exercise may be more desirable to concentrate on certain muscle groups or to avoid others. Thus, while muscle activation can be different, the kinematics and moments affecting the joints in the foot will remain constant regardless of stance. In addition, for both Earth-based and space-based exercise devices, it is important to build in flexibility that allows the subjects and workout program creators the opportunity to alter standard exercises to target specific muscles groups by altering foot stance when performing heel raises and squats.

## Limitations and future work

Our study has several limitations. First, the study utilized only one subject for a limited number of repetitions, which limits the power and generality of our conclusions. Future work should expand to more subjects completing a higher number of repetitions. In addition, future studies could also include a larger range of stance angles to assess and identify the dose response curve of foot stance to heel raise kinematics and dynamics. While our results indicated small variations in kinematics and dynamics when using different foot stances, additional study of muscle activation, and specifically the impact of the HULK device to muscle activation, will be desirable and advantageous to further characterize the performance of the HULK device. Finally, heel raises on the HULK device are performed with the load pulling the subject in front of the body and at the waist. The effect of this location compared to the load located on the subject’s back or in a seated position (e.g., on a leg press machine) will need to be addressed in future studies. Despite these limitations, the results of this study create a foundation that simultaneously supports future work and brings new insights into the development and applications of exercise protocols and countermeasures on Earth and in space.

## Data Availability

The raw data supporting the conclusion of this article will be made available by the authors, without undue reservation.

## Appendix

**TABLE A1:** Average (±SD) peak angle, range of motion, peak moment, and angular impulse during heel raises for inward foot stance, outward foot stance, straight foot stance, and straight stance without HULK conditions.

Joint	Stance	Peak angle (degrees)	Range of motion (degrees)	Peak moment (N*m)	Angular impulse (N × m × s)
Ankle	Inward foot stance	−32.8 (±0.7)	33.8 (±0.8)^b^ ^,^ ^c^	−92.5 (±4.3)	−68.1 (±6.0)
	Outward foot stance	−33.3 (±0.5)	30.8 (±0.9)^a^	−88.6 (±3.8)	−71.1 (±4.8)
	Straight foot stance	−31.7 (±1.2)	30.8 (±0.9)^a^	−86.4 (±3.2)	−65.3 (±4.1)
	Straight without HULK	−30.9 (±0.9)	33.4 (±0.2)^d^	−52.7 (±0.9)^d^	−43.4 (±4.7)^d^
MTP	Inward foot stance	−26.9 (±0.6)	24.7 (±1.0)	17.2 (±1.4)	8.2 (±0.5)^b^
	Outward foot stance	−26.2 (±0.6)	23.3 (±0.4)^c^	17.4 (±1.4)	10.2 (±1.1)^a^ ^,^ ^c^
	Straight foot stance	−26.8 (±0.4)	24.6 (±0.7)^b^	15.9 (±1.0)	7.8 (±1.1)^b^
	Straight without HULK	−23.4 (±1.3)^d^	20.9 (±0.4)^d^	10.9 (±1.8)^d^	7.2 (±2.3)
Subtalar	Inward foot stance	15.1 (±2.2)	5.4 (±1.2)	14.0 (±0.5)	6.3 (±0.9)
	Outward foot stance	12.5 (±2.0)	7.3 (±2.0)	15.8 (±0.9)	7.4 (±0.5)^c^
	Straight foot stance	16.4 (±3.0)	8.8 (±3.5)	14.5 (±0.9)	5.8 (±0.9)^b^
	Straight without HULK	9.8 (±1.5)^d^	6.0 (±0.9)	9.3 (±0.6)^d^	6.1 (±1.0)

a Statistical difference from inward foot stance (*α* = 0.05/3).

b Statistical difference from outward foot stance (*α* = 0.05/3).

c Statistical difference from straight foot stance (*α* = 0.05/3).

d Statistical difference between the straight foot stance and straight stance without HULK, condition (*α* = 0.05).
